# Impact of a brochure and empathetic physician communication on patients’ perception of breast biopsies

**DOI:** 10.1007/s00404-023-07058-w

**Published:** 2023-05-20

**Authors:** Martina Machacek, Corinne Urech, Sibil Tschudin, Laura Werlen, Cora-Ann Schoenenberger, Rosanna Zanetti-Dällenbach

**Affiliations:** 1 Department of Gynecology and Obstetrics, GZO Spital Wetzikon, Spitalstrasse 66, 8620 Wetzikon, Switzerland; 2 Department of Obstetrics and Gynecology, University Hospital Basel, Spitalstrasse 21, 4056 Basel, Switzerland; 3 Department of Clinical Research, University of Basel, University Hospital Basel, Spitalstrasse 12, 4031 Basel, Switzerland; 4 Department of Chemistry, University Basel, BioPark 1096, Mattenstrasse 24a, 4058 Basel, Switzerland; 5 Gynecology/Gynecologic Oncology, St.Claraspital Basel, Kleinriehenstrasse 30, 4002 Basel, Switzerland

**Keywords:** Anxiety, Breast biopsy, Breast cancer, STAI-S, STAI-T, Pain

## Abstract

**Purpose:**

This study investigated the effect of an intervention designed to reduce patients’ emotional distress associated with breast biopsy.

**Methods:**

125 breast biopsy patients receiving standard of care (control group, CG) were compared to 125 patients (intervention group, IG) who received a brochure with information prior to the biopsy and were biopsied by physicians trained in empathic communication. Anxiety was assessed by the State-Anxiety Inventory (STAI-S) at four time points (pre- and post-procedural, pre- and post-histology). All participants completed pre- and post-procedural questionnaires addressing worries, pain and comprehension. We evaluated the impact of the intervention on STAI-S levels using a log-transformed linear mixed effects model and explored patients’ and physicians’ perceptions of the procedure descriptively.

**Results:**

Post-procedural and post-histology timepoints were associated with 13% and17% lower with STAI-S levels than at the pre-procedural timepoint on average. The histologic result had the strongest association with STAI-S: malignancy was associated with 28% higher STAI-S scores than a benign finding on average. Across all time points, the intervention did not affect patient anxiety. Nevertheless, IG participants perceived less pain during the biopsy. Nearly all patients agreed that the brochure should be handed out prior to breast biopsy.

**Conclusion:**

While the distribution of an informative brochure and a physician trained in empathic communication did not reduce patient anxiety overall, we observed lower levels of worry and perceived pain regarding breast biopsy in the intervention group. The intervention seemed to improve patient’s understanding of the procedure. Moreover, professional training could increase physicians’ empathic communication skills.

**Trial registration number:**

NCT 02796612 (March 19, 2014).

**Supplementary Information:**

The online version contains supplementary material available at 10.1007/s00404-023-07058-w.

## What does this study add to the clinical work

**Table:** 

Handing out a brochure providing standardized information about the breast biopsy and supporting the patient by a physician trained in empathic communication can be associated with lower perceived pain during the biopsy, reduced post-procedural anxiety, and improved patient’s sense of being well informed.	

## Introduction

It is well documented that women experience high levels of psychological distress and anxiety during the diagnostic period of a suspicious breast lesion [10, 12, 25]. Breast biopsies elicit higher levels of anxiety in patients than other therapeutic interventions (hepatic chemoembolization, uterine fibroid embolization) [12] or elective surgery (cholecystectomy) [14], although the risk for complications and morbidity is lower. Reasons for anxiety include the concerns evoked by suspicious breast imaging, lack of understanding about why the procedure must be performed, the biopsy procedure itself, the expectation of pain and the fear of being diagnosed with breast cancer with all its consequences [15, 19, 23, 24].

Previous studies have shown that music [3], meditation [29], oral anxiolytic medication [6] and hypnosis [16] reduce patient’s anxiety. Empathic communication by physicians was shown to reduce anxiety in patients with advanced cancer [13]. However, there are no data regarding such an effect in the context of a breast biopsy as yet.

Hence, the primary aim of our study was to examine the impact of a brochure illustrating the breast biopsy procedure and information provided by a physician with specialized training in empathic communication on the patient’s anxiety. Furthermore, we explored whether patients diagnosed with breast cancer differ in their anxiety levels from women with benign breast lesions.

## Materials and methods

This prospective multicenter study was approved by the local ethical committee (Ethikkommission Nordwest- und Zentralschweiz; EKNZ 261/12) and was performed from March 2014 through September 2018. Study participants were required to provide written informed consent, understand and speak German and to be scheduled for a breast biopsy. Excluded were patients undergoing the procedure with fine needle aspiration (symptomatic cyst or abscess evacuation), male patients and patients younger than 18 years. We recruited 257 patients from three Swiss teaching hospitals of which seven were excluded due to consent withdrawal and unblinding. Thus, the final study population comprised 250 patients.

**Fig. 1:**
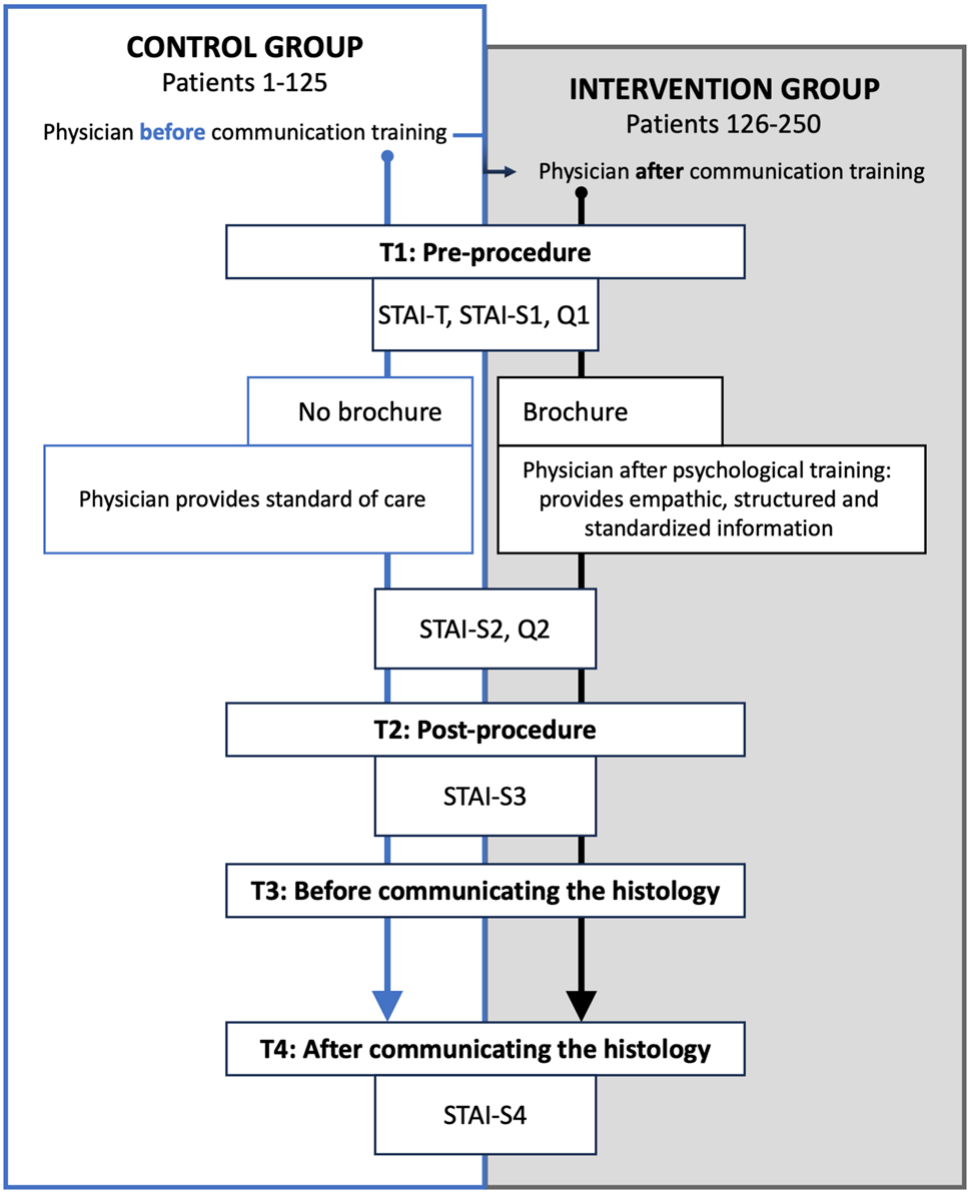
Outline of the sequential study design. T: timepoint; Q: questionnaire

To examine the effect of the communication training, a sequential study was designed (Fig. 1). The first 125 participants served as control group (CG) and were provided with the established standard of care, i.e., they were informed about the results of the clinical, mammographic and sonographic findings, and the breast biopsy procedure was explained to them by the physician performing it. Subsequently, the same physicians (n = 8) underwent a specialized one-day psychological training in small groups during which two psychologists taught them how to empathically provide structured and standardized information. This training addressed how to communicate details on the biopsy procedure and how to meet the patients’ emotional needs and particularly how to address patients’ anxiety regarding the biopsy and the fear associated with the possibility of breast cancer. The training involved role-playing with professional actors (simulation-patient) to efficiently mimic real patient-physician interactions.

Subsequently, patients were recruited for participation in the intervention group (IG). They received information on the biopsy procedure by the psychologically trained physicians and a brochure (included as supplemental material) which explained the breast biopsy procedure in detail.

To assess anxiety associated with the breast biopsy, all participants filled out the German version of the validated State-Trait Anxiety Inventory (STAI) [30]. STAI-trait (STAI-T) measures the general anxiety of an individual, which is assumed not to change over time and is henceforth considered a patient characteristic. STAI-state (STAI-S) which measures the level of anxiety at a given time point (T) in reaction to a specific event, was assessed pre- (T1) and post-procedural (T2), and pre- (T3) and post-histology (T4) i.e. before and after communicating the result of the biopsy. Both, STAI-T and STAI-S consist of 20 self-rating items rated on 4-point Likert scales, and yield a total score between 20 and 80. The higher the score, the higher the level of anxiety.

All study participants completed a pre- (Q1) and post-procedural (Q2) questionnaire (see Supplementary material) addressing their worries and comprehension regarding the breast biopsy and the level of anticipated and experienced pain. During the biopsy procedure, the study nurses asked the patients to rate their worries and levels of anticipated pain on a visual-analogue-scale (VAS) (see Supplementary material). After the biopsy procedure, the physicians also rated the patient’s worries, pain and satisfaction with the procedure on a VAS (see Supplementary material).

Patient characteristics, personal and family history as well as mammographic and sonographic breast density were obtained from the electronic patient chart (ViewPoint®, Version 5: GE Healthcare GmbH).

### Statistical analyses

All analyses were conducted in the statistical software environment R version 4.0.3 (R Core Team, 2020). We summarized the patients’ characteristics overall and by study group. In addition, we present the summary statistics of the STAI-S scores at the four different time points as well as for the patient and physician questionnaires at each time point by study group and histological result. To evaluate the impact of the intervention on STAI-S, we used a log-transformed linear mixed effects model with the intervention condition as a predictor and the natural log of STAI-S as the outcome using the package lme4 [2]. We log-transformed the outcome due to its skewed distribution, but transformed estimated coefficients back to the original scale of STAI-S in the presented results. The results shown thus represent multiplicative effects. Patients were included in the model as a random effect. The model was adjusted for the following covariates: time point, patient’s age, educational level, sonographic breast density [18] family or personal history of cancer, trait anxiety score (measured pre-biopsy), time elapsed between biopsy and receiving the results of the biopsy, and result of the biopsy. The estimated effect of the intervention thus controls for these variables. We included an interaction between assignment to condition and time point in the model.

## Results

Patient and lesion characteristics are summarized by study group in Table 1 and by study site in Table S1 (see Supplementary material).

**Table 1:** Patients characteristics

	Study population (n = 250)	Control group (n = 125)	Intervention group (n = 125)
**Age** in years (mean) [SD] (min, max)	51.4 [18.2] (18, 90)	49.5 [16.9] (18, 87)	53.3 [19.2] (18, 90)
**STAI-T** (median) [IQR]	40.0 [35.00, 46.00]	40.0 [35.00, 46.00]	39.0 [34.00, 44.00]
**Educational status** n (%)			
Compulsory education	25 (10.0)	12 (9.6)	13 (10.4)
Vocational training	112 (44.8)	57 (45.6)	55 (44.0)
University	105 (42.0)	51 (40.8)	54 (43.2)
Not specified	8 (3.2)	5 (4.0)	3 (2.4)
**Civil status** n (%)			
Single	100 (40.0)	46 (36.8)	54 (43.2)
Married / in partnership	149 (59.6)	79 (63.2)	70 (56.0)
Not specified	1 (0.4)	0 (0.0)	1 (0.8)
**Number of children** (median) (min, max) [IQR]	1 (0, 5) [0.0, 2.0]	1 (0, 4) [0.0, 2.0]	1 (0, 5) [0.0, 2.0]
**Personal history of breast biopsy** n (%)			
No	196 (78.4)	89 (71.2)	107 (85.6)
Yes	53 (21.2)	36 (28.8)	17 (13.6)
Unknown	1 (0.4)	0 (0.0)	1 (0.8)
**Personal history of breast operation with benign histology** n (%)			
No	226 (90.4)	114 (91.2)	112 (89.6)
Yes	23 (9.2)	11 (8.8)	12 (9.6)
Unknown	1 (0.4)	0 (0.0)	1 (0.8)
**Personal history of breast cancer** n (%)			
No	236 (94.4)	117 (93.6)	119 (95.2)
Yes	13 (5.2)	8 (6.4)	5 (4.0)
Unknown	1 (0.4)	0 (0.0)	1 (0.8)
**Family history of breast cancer** n (%)			
No	167 (66.8)	74 (59.2)	93 (74.4)
Yes	76 (30.4)	47 (37.6)	29 (23.2)
Unknown	7 (2.8)	4 (3.2)	3 (2.4)
Sonographic breast density* n (%)			
1	23 (9.2)	10 (8.0)	13 (10.4)
2	79 (31.6)	35 (28.0)	44 (35.2)
3	113 (45.2)	62 (49.6)	51 (40.8)
4	34 (13.6)	17 (13.6)	17 (13.6)
Not specified	1 (0.4)	1 (0.8)	0 (0.0)
**Waiting time for biopsy results in days**			
(median) [IQR]	7.0 [3.1, 8.0]	6.8 [2.9, 7.9]	7.0 [6.0, 9.0]
**Histologic diagnosis** n (%)			
Benign	149 (59.6)	79 (63.2)	70 (56.0)
Malignant	101 (40.4)	46 (36.8)	55 (44.0)

*According to Madjar et al. 2006

CG and IG appeared to be comparable with regard to most variables of interest (i.e. mean age, STAI-T) with the exception of personal or family history of breast cancer [9]. In the CG 28.8% of the participants had experienced a breast biopsy in the past as opposed to 13.6% in the IG. In the CG, 37.6% had a positive family history compared to 23.2% the IG.

Overall, BC was diagnosed in 101 (40.4%) women, while 149 (59.6%) women had a benign breast lesion (BBL). More women were diagnosed with BC in the IG (n = 55, 44.0%) versus the CG (n = 46, 36.8%). STAI-T values were comparable in patients with BBL [40.0 (IQR 35.0, 46.0)] and BC patients ]39.0 (IQR 33.0, 47.0)].

**Fig. 2:**
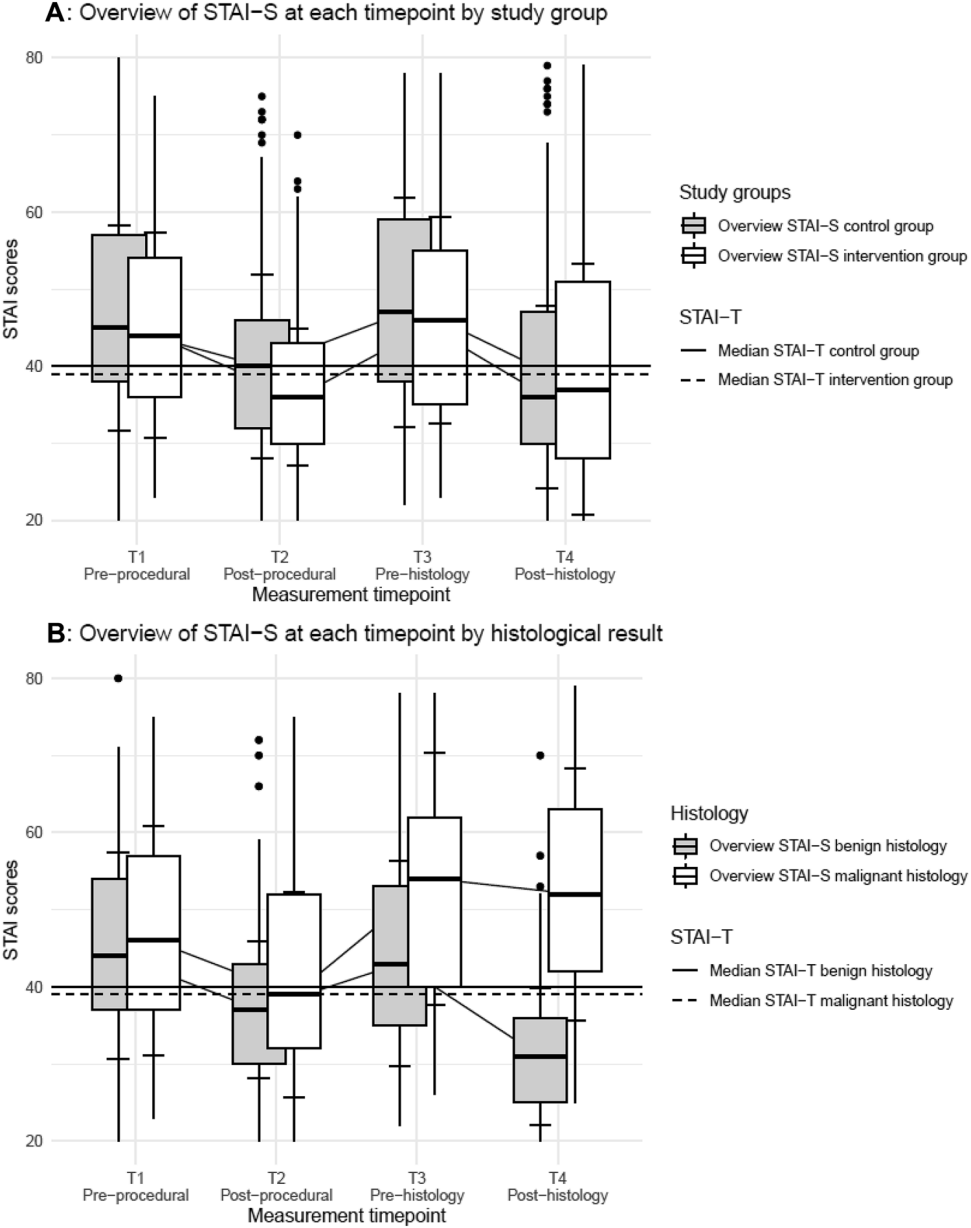
STAI-S at four different time points in **A** CG versus IG, **B** Benign versus malignant histology

Patient anxiety was assessed by STAI-S at four different time points: immediately pre-(T1) and post-procedural (T2); immediately pre-(T3) and post-histology (T3). Over all time points, we did not see an association between the intervention and STAI-S scores (point estimate 2% lower scores in the intervention group compared to the control group, 95% CI between 8% lower and 5% higher). With regard to the entire study population, we observed that the post-procedural (T2) and post-histology (T4) STAI-S scores were lower than pre-procedural (T1) STAI-S scores (13% and 17%, respectively) (Table 2). Furthermore, we observed large differences in STAI-S by histologic result at time points 3 and 4: patients with BC had 28% higher STAI-S scores than patients with BBL. The results of the STAI-S scores for CG and IG at the four different time points are summarized in Fig. 2 and Table 3.

**Table 2:** Analysis of STAI-S by time point testing for an interaction between time and assigned condition

	Estimate	CI (95% lower, 95% upper)
**Intervention group vs. control group**	0.98	(0.92, 1.05)
**Time points**		
Time point 2 versus time point 1	0.87	(0.83, 0.92)
Time point 3 versus time point 1	1.03	(0.98, 1.09)
Time point 4 versus time point 1	0.83	(0.79, 0.88)
**Interaction between the study group and the different time points**		
Intervention group at time point 2 (vs. control group at time point 1)	0.94	(0.88, 1.02)
Intervention group at time point 3 (vs. control group at time point 1)	0.97	(0.90, 1.05)
Intervention group at time point 4 (vs. control group at time point 1)	1.01	(0.94, 1.09)
**Confounders**		
Age (in years)	1.00	(1.00, 1.00)
STAI-T	1.01	(1.01, 1.01)
Educational status		
Vocational training versus no vocational training	1.04	(0.96, 1.14)
University versus no vocational training	1.03	(0.94, 1.12)
Not specified versus no vocational training	0.99	(0.85, 1.16)
Family or personal history of cancer		
Positive family or personal history versus negative family or personal history	1.02	(0.97, 1.08)
Unknown family or personal history versus negative family or personal history	0.93	(0.80, 1.08)
Sonographic breast density		
2 versus 1	1.03	(0.94, 1.13)
3 versus 1	1.06	(0.96, 1.16)
4 versus 1	1.05	(0.94, 1.18)
Waiting time for the biopsy results in days (in days)	1.0	(0.99, 1.00)
Malignant versus benign histology	1.28	(1.21, 1.36)

**Table 3:** STAI-S at the four different timepoints

	Study population	Control group	Intervention group	Benign histology	Malignant histology
	n = 250	n = 125	n = 125	n = 149	n = 101
**STAI-S at the different time points**					
STAI-S T1 (median) [IQR]	44.5 [37.0, 56.0]	45.0 [38.0, 57.0]	44.0 [36.0, 54.0]	44.0 [37.0, 54.0]	46.0 [37.0, 57.0]
STAI-S T2 (median) [IQR]	38.0 [31.0, 46.0]	40.0 [32.0, 46.0]	36.0 [30.0, 43.0]	37.0 [30.0, 43.0]	39.0 [32.0, 52.0]
STAI-S T3 (median) [IQR]	46.5 [37.0, 57.0]	47.0 [38.0, 59.0]	46.0 [35.0, 55.0]	43.0 [35.0, 53.0]	54.0 [40.0, 62.0]
STAI-S T4 (median) [IQR]	36.0 [28.0, 49.8]	36.0 [30.0, 47.0]	37.0 [28.0, 51.0]	31.0 [25.0, 36.0]	52.0 [42.0, 63.0]

Worries (measured by VAS) during the biopsy procedure were lower in the IG compared to CG (Table 4). Notably, immediately after the biopsy, patients who turned out to have BC were more worried than those with a BBL.

**Table 4:** Patient worries

	Study population	Control group	Intervention group	Benign histology	Malignant histology
	n = 250	n = 125	n = 125	n = 149	n = 101
Timepoint 1, VAS*					
I am scared of the biopsy procedure (median) [IQR]	4.0 [2.0, 6.0]	4.0 [2.0, 6.0]	4.0 [1.0, 5.0]	4.0 [2.0, 6.0]	4.0 [2.0, 6.0]
I am scared of the biopsy outcome (median) [IQR]	5.0 [3.0, 7.0]	5.0 [3.0, 7.0]	5.0 [3.0, 7.0]	5.0 [3.00, 7.00]	5.0 [2.0, 7.0]
Biopsy associated worries, VAS**					
Immediately before biopsy (median) [IQR]	4.0 [2.0, 5.0]	4.0 [3.0, 6.0]	3.0 [2.0, 4.0]	3.0 [2.0, 5.0]	4.0 [2.0, 5.0]
During the biopsy (median) [IQR]	4.0 [2.0, 5.0]	4.0 [3.0, 6.0]	3.0 [1.0, 4.0]	3.0 [2.0, 5.0]	4.0 [2.0, 5.0]
Immediately after biopsy (median) [IQR]	2.0 [1.0, 3.0]	2.0 [1.0, 4.0]	2.0 [0.0, 3.0]	2.0 [0.0, 3.0]	2.0 [1.0, 3.0]
**Timepoint 2**					
Biopsy was far easier than expected n (%)	51 (20.4)	0 (0.0)	51 (40.8)	37 (24.8)	14 (13.9)
Biopsy was easier than expected n (%)	75 (30.0)	36 (28.8)	39 (31.2)	40 (26.8)	35 (34.7)
Biopsy was as expected n (%)	76 (30.4)	48 (38.4)	28 (22.4)	45 (30.2)	31 (30.7)
Biopsy worse n (%)	9 (3.6)	4 (3.2)	5 (4.0)	5 (3.4)	4 (4.0)
Biopsy was far worse n (%)	3 (1.2)	1 (0.8)	2 (1.6)	0 (0.0)	3 (3.0)
No answer n (%)	36 (14.4)	36 (28.8)	0 (0.0)	22 (14.8)	14 (13.9)

*Visual analogue scale (VAS): 0: no worries; 10: maximal worries

**Immediately before, during and, directly after the biopsy procedure the study nurse asked the patient about her worries on a VAS from 0 to 10: 0: no worries; 10: maximal worries

The post-procedural questionnaire showed that 72% from the IG and 28.8% from the CG rated the biopsy easier or far easier than expected (Table 4) This rating was similar for patients with and without BC.

The pain perceived immediately before and immediately after the biopsy was less in the IG (median VAS 1) than in the CG (median VAS 2) (Table 5). In comparison to women with BBL, BC patients perceived more pain during and immediately after the biopsy.

**Table 5:** Patient perception of pain associated with the biopsy procedure

	Study population	Control group	Intervention group	Benign histology	Malignant histology
	n = 250	n = 125	n = 125	n = 149	n = 101
Timepoint 1, VAS*					
I expect the biopsy to be painful (median) [IQR]	4.0 [2.0, 5.0]	4.0 [2.0, 5.0]	4.0 [2.0, 5.0]	4.00 [2.00, 5.00]	4.00 [2.00, 5.00]
Biopsy related pain, VAS**					
Immediately before biopsy (median) [IQR]	1.0 [0.0, 2.0]	2.0 [1.0, 2.0]	1.0 [0.0, 2.0]	1.0 [0.0, 2.0]	2.0 [0.0, 2.0]
During biopsy (median) [IQR]	2.0 [1.0, 3.0]	2.0 [1.0, 3.0]	2.0 [1.0, 3.0]	2.0 [1.0, 3.0]	3.0 [2.0, 4.0]
Immediately after biopsy (median) [IQR]	1.0 [0.0, 2.0]	2.0 [1.0, 2.0]	1.0 [0.0, 2.0]	1.0 [0.0, 2.0]	2.0 [1.0, 2.0]
**Timepoint 2**					
I had no pain n (%)	173 (69.2)	81 (64.8)	92 (73.6)	108 (72.5)	65 (64.4)
I had pain n (%)	77 (30.8)	44 (35.2)	33 (26.4)	41 (27.5)	36 (35.6)
Far less pain than expected n (%)	23 (9.2)	14 (11.2)	9 (7.2)	18 (12.1)	5 (5.0)
Less pain than expected n (%)	16 (6.4)	9 (7.2)	7 (5.6)	7 (4.7)	9 (8.9)
As expected n (%)	23 (9.2)	14 (11.2)	9 (7.2)	12 (8.1)	11 (10.9)
Greater than expected n (%)	12 (4.8)	7 (5.6)	5 (4.0)	4 (2.7)	8 (7.9)
Much greater than expected n (%)	3 (1.2)	0 (0.0)	3 (2.4)	0 (0.0)	3 (3.0)

*Visual analogue scale (VAS): 0: no pain; 10: maximal pain

** Immediately before, during and, directly after the biopsy procedure the study nurse asked the patient about her pain on a VAS from 0 to 10: 0: no pain; 10: maximal pain

Regarding the comprehension of the upcoming biopsy procedure, patients from the IG reported feeling better informed than patients in the CG (Table 6). Similarly, physicians who performed the biopsy perceived the IG as less worried and consequently more satisfied with the procedure than those from the CG (Supplementary material, Table S2).

**Table 6:** Patient comprehension

	Study population	Control group	Intervention group	Benign histology	Malignant histology
	n = 250	n = 125	n = 125	n = 149	n = 101
Timepoint 1, VAS*					
I understand the necessity of the biopsy (median) [IQR]	1.0 [0.0,1.0]	1.0 [0.0, 2.0]	1.0 [0.0, 1.0]	1.0 [0.0, 1.0]	1.0 [0.0, 2.0]
I am well informed about the procedure (median) [IQR]	1.0 [0.0, 2.0]	1.0 [1.0, 3.0]	1.0 [0.0, 2.0]	1.0 [0.0, 2.0]	1.0 [0.0, 2.0]
Timepoint 2, VAS*					
The necessity of the biopsy is clear to me (median) [IQR]	1.0 [0.0, 1.0]	1.0 [0.0, 1.0]	1.0 [0.0, 1.0]	1.0 [0.0, 1.0]	1.0 [1.0, 1.0]
I was well informed about the procedure (median) [IQR]	1.0 [0.0, 2.0]	1.0 [0.0, 2.0]	1.0 [0.0, 2.0]	1.0 [0.0, 2.0]	1.0 [1.0, 2.0]
I received clear information by the physician (median) [IQR]	1.0 [0.0, 2.0]	1.0 [0.0, 2.0]	1.0 [0.0, 2.0]	1.0 [0.0, 2.0]	1.0 [1.0, 2.0]

*Visual analogue scale (VAS): 0: excellent information; 10: poor information

Based on the post-procedural questionnaire (T2), 54.4% of the CG thought that a brochure explaining the breast biopsy procedure would be helpful (Supplementary material, Table S3). In fact, the median VAS rating for a brochure was 2 ([IQR 1.0, 3.0]), with 0 being extremely helpful and 10 not helpful at all. 95.2% of the IG agreed that the brochure should be handed out to all patients requiring a breast biopsy [VAS of 2, (IQR 1.0, 3.0)].

## Discussion

The lower STAI-S at T2 compared to T1 for both the CG and the IG suggests that having completed the biopsy reduces anxiety. More specifically, our data show that post-procedural (T2) anxiety was reduced to a larger extent in the IG, which had received a brochure and support during the diagnostic procedure by a physician trained in empathic communication. In line with the intervention, patients in the IG felt they were well informed. Moreover, compared to the CG, patients of the IG perceived less pain during the biopsy. Nearly all patients agreed that a brochure should be distributed to all patients requiring a breast biopsy although considering all 4 timepoints of anxiety assessment (T1–T4), an impact of handing out a brochure and physician support on patient anxiety was not apparent.

Our data collected at four time points suggest that the diagnostic period has a defined dynamic trajectory of anxiety (Fig. 2a). Overall, we did not see major differences in STAI-S between the CG and the IG. Consistent with previous reports [3, 6, 19, 22, 29], our study shows that post-procedural (T2) anxiety is generally lower than pre-procedural anxiety. It is possible that the intervention has the most potential to impact anxiety at this time point. Studies investigating other anxiety-reducing interventions, including listening to music [3] or guided meditation [29], also showed a decrease in post-procedural anxiety compared to a control group.

The dynamic trajectory of anxiety (Fig. 2b) in women with diagnosed with BC revealed higher STAI-S values at T2, T3 and T4 compared to women with BBL. Similarly, Maimone et al. and Novy et al. reported higher STAI-S in BC patients [19, 25]. Of note is the pre-histology anxiety (T3) in particular, which was higher in BC patients (median STAI-S 54.0) compared to women with BBL (median STAI-S 43.0). This could indicate that patients had a premonition of having BC. Consistent with this notion, Poole et al. [26] report that in a group of patients with high anxiety (mean STAI-S 67.46), 71.4% had BC.

As we anticipated and was shown by others [21], the post-histology anxiety in women diagnosed with BC (median STAI-S 52.0) was higher compared to women with BBL (median STAI-S 41.0). The large STAI-S drop in women with BBL could reflect the relief of not being diagnosed with BC.

The patients’ worries immediately before, during and immediately after the biopsy were lower in the IG compared to the CG. The worries did not differ between women with BBL and BC, most likely because women do not know the histology of their breast lesion at this time point. An anxiolytic medication-related reduction in self-reported anxiety during the procedure was reported by Bugbee and coworkers, but was not evident before and 24 h post-procedure [6]. Besides the differences in the intervention, the longtime interval between procedure and reporting, does not allow for a direct comparison of their study and ours.

The median VAS for anticipated pain was 4 in both groups, which is in line with previously reported findings [19, 28]. The pain perceived during and after the biopsy ranged from VAS 2–3 during and from VAS 1–2 after the biopsy. Similarly, other studies report pain perception ranging from VAS 1.25 [28]–VAS 2.3 [27] during, and VAS 1.3 [21] immediately after the biopsy.

Immediately before and immediately after the biopsy the IG perceived less pain than the CG, suggesting that the intervention could have a positive impact on pain perception. The results were similar to those from studies of other interventions with the aim to reduce anxiety. For example, patients receiving structured empathic attention or performing self-hypnotic relaxation were reported to perceive less pain than patients receiving standard care [16]. Furthermore, patients listening to music were found to experience less pain than patients listening to guided meditation or receiving standard of care [29]. Brief mindfulness interventions on the other hand did not lead to reduction of pain [8]. While our data suggest a difference in perceived pain between women with BBL and BC, others found no difference between these groups [19, 27, 28].

All participants rated their comprehension of the procedure to be excellent. This data is in line with Brandon et al. [5] who reported that 94% of patients perceive explanations regarding the indication of the biopsy satisfactory and 99% found the information about the procedure itself adequate.

It is well accepted that an illustrated brochure explaining a procedure is an effective tool to provide standardized information [7, 19, 20]. Consistent with this notion, 54.4% of the CG stated that a brochure would be helpful and 95.2% of the IG rated the brochure to be helpful. This is in line with the data of Maimone et al. [19] where 87.0% valued a corresponding brochure to be beneficial.

Most physicians are not specially trained in communication [1, 11], but they are experienced in discussing diagnosis and procedures. However, this might not meet the patient’s emotional needs [17]. Empathic communication reduces emotional distress as well as pre- and post-procedural anxiety [22] and thus, increases patient satisfaction [4, 13]. Overall, high patient satisfaction scores are closely related to the information provided by physicians [4].

Our study included a large number of participants (n = 250) compared to the majority of studies in the literature. A further strength of our study is that it provides data over the entire diagnostic period, from the pre-procedure (T1) to the post-histology (T4) time point. However, our study faced an important limitation. To rule out that differences in the outcome of our study are related to different physicians, the IG was treated by the same physician as CG. This sequential study design made randomization to study condition and blinding unfeasible.

## Conclusion

Handing out a brochure and supporting the patient during the diagnostic procedure by a physician trained in empathic communication could be associated with lower perceived pain during the biopsy and reducing post-procedural anxiety. In addition, the intervention could positively affect patient’s sense of being well informed about the procedure. Moreover, the communication training appears to increase the physicians’ empathic communications skills. We conclude that a brochure providing standardized information about the breast biopsy procedure is helpful, and is now handed out to all our patients requiring a breast biopsy.

### Supplementary Information

Supplementary file 1: (PDF 621 kb)

Supplementary file 2: (PDF 71 kb)

Supplementary file 3: (PDF 96 kb)

Supplementary file 4: (PDF 91 kb)

Supplementary file 5: (DOCX 18 kb)

Supplementary file 6: (DOCX 16 kb)

Supplementary file 7: (DOCX 16 kb)

## Data Availability

The Data are available upon reasonable request to the corresponding author.
