# Supplementary material for: Impact of a brochure and empathetic physician communication on patients’ perception of breast biopsies
Source: Arch Gynecol Obstet. 2023 May 20;308(5):1611–20. doi: 10.1007/s00404-023-07058-w (PMC10520099; doi:10.1007/s00404-023-07058-w)
Supplement: Supplementary file 1 — (PDF 621 kb) [file 404_2023_7058_MOESM1_ESM.pdf]

Im Rahmen der Brustuntersuchung, des Brustultraschalls und/oder der Mammographie wurde bei Ihnen eine Veränderung in der Brust festgestellt. Eine Gewebeprobe (Brustbiopsie) soll nun klären, ob es sich um eine gutartige oder bösartige Veränderung handelt.

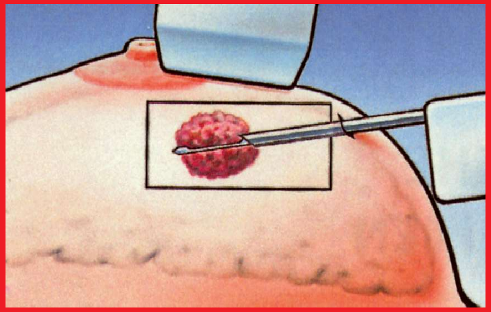

unter Ultraschallkontrolle
durchgeführte Brustbiopsie

Informationen zur Entnahme einer Gewebeprobe aus der Brust (Brustbiopsie)

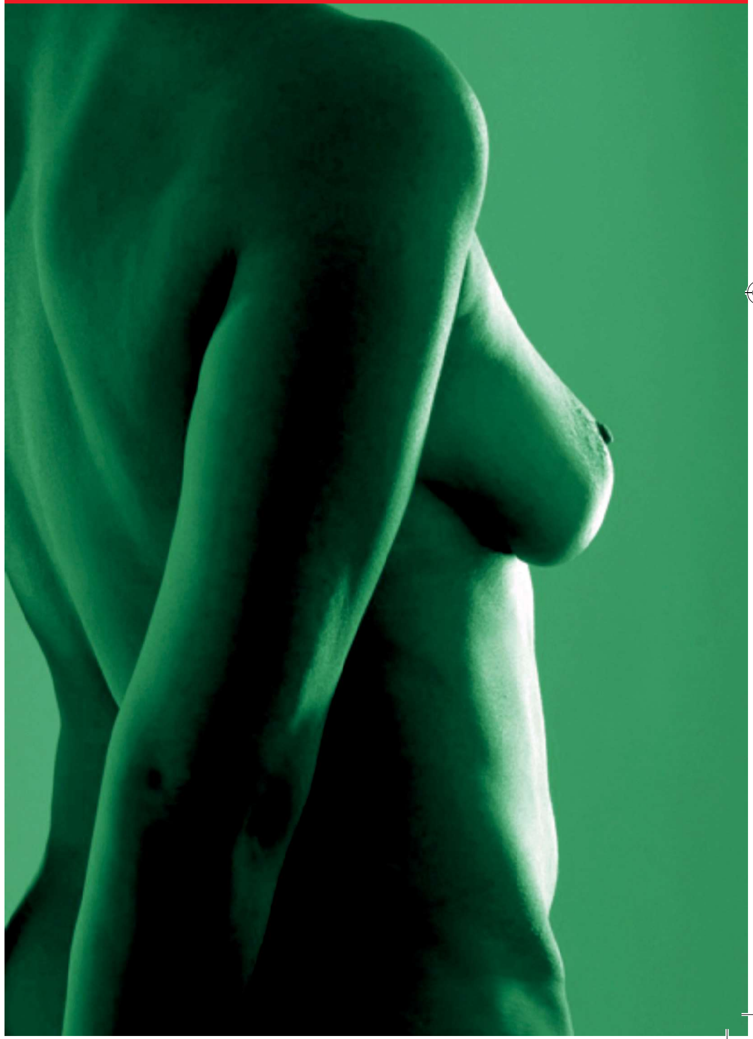

An welcher Stelle wird die Biopsie entnommen?

- Die Biopsie wird unter sonographischer Kontrolle aus der beobachteten Veränderung entnommen.

Wie sieht das Vorgehen aus?

- Bei der Brustbiopsie liegen Sie wie bei einer Ultraschalluntersuchung auf dem Rücken. Es ist uns ein Anliegen, dass Sie bequem liegen. Bitte teilen Sie uns mit, wenn Sie Gelenk- oder Muskelschmerzen haben, damit wir Sie mit Hilfe von Kissen und Tüchern möglichst schmerzfrei lagern können.
- Die Veränderung in der Brust wird zuerst mit dem Ultraschall lokalisiert.
- Nach Desinfektion der Haut wird eine örtliche Betäubung (Lokalanästhesie) durchgeführt. Bitte teilen Sie uns mit, falls Sie dennoch Schmerzen haben.
- Die Biopsienadel wird unter Ultraschallsicht durch einen kleinen Hautschnitt (2,5 bis 5 mm) bis an die Veränderung gebracht, um dann mehrere Gewebeproben (meistens fünf) aus der Veränderung zu entnehmen.
- Gelegentlich ist es notwendig, die betroffene Stelle nach der Gewebeprobe mit einem Clip in der Brust zu markieren. So kann der Entnahmeort jederzeit wieder lokalisiert und kontrolliert werden. Jede Brustbiopsie wird genau dokumentiert.
- Der kleine Hautschnitt wird mit schmalen Pflastern versorgt. Zusätzlich wird ein Druckverband angelegt, um einen Bluterguss oder eine Blutung möglichst zu vermeiden.
- Bei Bedarf können wir Ihnen Schmerzmedikamente mitgeben.

Was geschieht mit der Gewebeprobe?

- Die entnommene Gewebeprobe wird ins Labor geschickt und dort unter dem Mikroskop untersucht.
- In der Regel liegt nach 3 bis 5 Arbeitstagen das Ergebnis der Gewebeprobe vor, welches wir dann mit Ihnen besprechen.

Wie muss ich mich nach der Brustbiopsie verhalten?

- Am Tag der Brustbiopsie sollten Sie auf sportliche Aktivitäten sowie auf extreme körperliche Anstrengungen verzichten. Ansonsten müssen Sie sich nicht weiter schonen.
- Sollten Sie berufstätig sein, ist es je nach Beruf sinnvoll, am Tag der Biopsie nicht zur Arbeit zu gehen. Bitte teilen Sie uns mit, wenn Sie ein Arbeitsunfähigkeitszeugnis benötigen.
- Der Druckverband wird idealerweise für 24 Stunden belassen. Die schmalen Pflaster, mit welchen Sie duschen und baden können, dürfen Sie nach 7 Tagen entfernen.
- In der Regel verheilt der kleine Hautschnitt mit einer kaum wahrnehmbaren Narbe.

Welche Komplikationen und Nebenwirkungen können auftreten?

- Gewöhnlich verläuft eine Brustbiopsie völlig komplikationslos.
- Beim Einführen der Nadel und während der Biopsie können kleine Blutgefässe verletzt werden. Dadurch kann es zu Blutungen, blauen Flecken und Blutergüssen kommen.
- Selten werden – trotz sorgfältigster Punktionstechnik – bei sehr kleinen Veränderungen diese verfehlt oder das entnommene Gewebe reicht nicht aus, um eine klare Diagnose stellen zu können. In beiden Fällen muss die Biopsie wiederholt werden.
- Falls Sie zu einer übermässigen Narbenbildung (Keloid) neigen, kann auch ein sehr kleiner Hautschnitt zu einer übermässigen Narbenbildung und einer kosmetischen Beeinträchtigung führen.
- Andere Komplikationen sind sehr selten, wie z.B. Entzündungen, Wundheilungsstörungen, allergische Reaktionen auf die örtliche Betäubung und Verletzungen der Haut, des Brustmuskels oder der Nachbarorgane.

Die Resultate der Brustbiopsie liegen vor. Was geschieht jetzt?

- Im Falle einer gutartigen Diagnose ist keine Therapie notwendig. In der Regel empfehlen wir eine Kontrolluntersuchung mittels Ultraschall oder Mammographie (nach 6 bis 12 Monaten).
- Bei einem bösartigen Befund, d.h. bei Brustkrebs, werden die weiteren Behandlungsschritte wie Operation, medikamentöse Therapie und/ oder Strahlentherapie ausführlich mit Ihnen und Ihren Angehörigen besprochen.

Wer bezahlt die Brustbiopsie?

- Die Brustbiopsie ist eine Pflichtleistung der Krankenkasse.

Haben Sie weitere Fragen oder ist Ihnen etwas unklar?

- Bitte wenden Sie sich an Ihre betreuende Ärztin/ Ihren betreuenden Arzt, an eine Pflegefachperson oder an eine unserer Breast Care Nurses.

Ihr Studien-Team
