# Supplementary material for: Impact of a brochure and empathetic physician communication on patients’ perception of breast biopsies
Source: Arch Gynecol Obstet. 2023 May 20;308(5):1611–20. doi: 10.1007/s00404-023-07058-w (PMC10520099; doi:10.1007/s00404-023-07058-w)
Supplement: Supplementary file 2 — (PDF 71 kb) [file 404_2023_7058_MOESM2_ESM.pdf]

Pre-biopsy-questionnaire (Q1)

Fragebogen vor Biopsie

A Ich habe Angst vor der Biopsie.

überhaupt keine Angst *extrem grosse Angst*

B Ich erwarte Schmerzen bei der Biopsie.

überhaupt keine Schmerzen *unerträgliche Schmerzen*

C Ich fürchte mich vor der Diagnose.

überhaupt keine Angst *extrem grosse Angst*

D Ich denke das Resultat der Gewebeuntersuchung wird sein:

- □ gutartig
- □ bösartig
- □ weiss nicht
- □ möchte nicht antworten

E Mir ist klar, warum die Brust-Biopsie gemacht werden muss.

mir ist alles klar *Ich kann nicht verstehen, warum die Biopsie gemacht werden muss.*

F Ich wurde gut über das Vorgehen bei der Biopsie informiert.

sehr gut informiert *sehr schlecht informiert*

G Möchten Sie nach Abschluss der Studie über die Ergebnisse informiert werden?

- □ ja
- □ nein
