# Supplementary material for: Impact of a brochure and empathetic physician communication on patients’ perception of breast biopsies
Source: Arch Gynecol Obstet. 2023 May 20;308(5):1611–20. doi: 10.1007/s00404-023-07058-w (PMC10520099; doi:10.1007/s00404-023-07058-w)
Supplement: Supplementary file 3 — (PDF 96 kb) [file 404_2023_7058_MOESM3_ESM.pdf]

Post-biopsy-questionnaire (Q2-CG and Q2-IG)

Fragebogen nach Biopsie

A Wie haben Sie die Biopsie empfunden?

❒ viel weniger schlimm
❒ weniger schlimm
❒ wie erwartet
❒ schlimmer
❒ war viel schlimmer

B Wie stark waren die Schmerzen?

❒ ich hatte keine Schmerzen
❒ ich hatte Schmerzen

wenn Sie Schmerzen gehabt haben

❒ eindeutig weniger stark als erwartet
❒ weniger stark
❒ wie erwartet
❒ stärker als erwartet
❒ viel stärker als erwartet

C Mir ist klar, warum die Brust-Biopsie gemacht werden musste.

mir ist alles klar ——— *Ich kann nicht verstehen, warum die Biopsie gemacht werden musste.*

D Ich wurde gut über das Vorgehen bei der Biopsie informiert.

sehr gut informiert ——— *sehr schlecht informiert*

E Der Arzt / die Ärztin hat mich gut informiert.

sehr gut informiert ——— *sehr schlecht informiert*

CONTROL GROUP (Q2-CG)

F Sollte eine Broschüre mit Erklärungen zur Brustbiopsie abgegeben werden?

❒ wäre hilfreich
❒ unnötig
❒ weiss nicht
❒ möchte nicht antworten

extrem hilfreich ——— *überhaupt nicht hilfreich*

INTERVENTION GROUP (Q2-IG)

F Die Informations-Broschüre zur Brustbiopsie ist hilfreich.

❒ bitte weiterhin abgeben
❒ unnötig

extrem hilfreich ——— *überhaupt nicht hilfreich*
