# Supplementary material for: Impact of a brochure and empathetic physician communication on patients’ perception of breast biopsies
Source: Arch Gynecol Obstet. 2023 May 20;308(5):1611–20. doi: 10.1007/s00404-023-07058-w (PMC10520099; doi:10.1007/s00404-023-07058-w)
Supplement: Supplementary file 4 — (PDF 91 kb) [file 404_2023_7058_MOESM4_ESM.pdf]

VAS during the biopsy procedure

Zu erheben durch die Study Nurse

A Unmittelbar vor Beginn der Brustbiopsie?

Überhaupt nicht ängstlich = 0 10 = extrem ängstlich

Angabe der Patientin:_____

Überhaupt keine Schmerzen = 0 10 = sehr starke Schmerzen

Angabe der Patientin:______

Während der Brustbiopsie?

Überhaupt nicht ängstlich = 0 10 = extrem ängstlich

Angabe der Patientin:_____

Überhaupt keine Schmerzen = 0 10 = sehr starke Schmerzen

Angabe der Patientin:______

C Unmittelbar nach der Brustbiopsie?

Überhaupt nicht ängstlich = 0 10 = extrem ängstlich

Angabe der Patientin:_____

Überhaupt keine Schmerzen = 0 10 = sehr starke Schmerzen

Angabe der Patientin:______

Questionnaire for the physician

Fragebogen für Arzt/Ärztin

A Wie erleben Sie die Patientin?

überhaupt nicht ängstlich *extrem ängstlich*

überhaupt nicht angespannt *extrem angespannt*

überhaupt keine Schmerzen *sehr starke Schmerzen*

B Ist die Patientin mit der Intervention zufrieden?

äusserst zufrieden *gar nicht zufrieden*

Visum Arzt / Ärztin
