# Supplementary material for: Impact of a brochure and empathetic physician communication on patients’ perception of breast biopsies
Source: Arch Gynecol Obstet. 2023 May 20;308(5):1611–20. doi: 10.1007/s00404-023-07058-w (PMC10520099; doi:10.1007/s00404-023-07058-w)
Supplement: Supplementary file 5 — (DOCX 18 kb) [file 404_2023_7058_MOESM5_ESM.docx]

**Table S1**: Patient characteristics by centre

|  | **Study population**  **(n = 250)** | **Control Group**  **(n = 125)** | **Intervention Group (USB)**  **(n = 57)** | **Intervention Group (Claraspital)**  **(n = 35)** | **Intervention Group (GZO)**  **(n = 33)** |
| --- | --- | --- | --- | --- | --- |
|  |  |  |  |  |  |
| **Age** in years (mean)  [SD] (min, max) | 51.4  [18.2] (18, 90) | 49.5  [16.9] (18, 87) | 52.9  [19.0] (20, 90) | 53.5  [20] (18, 85) | 53.8  [19.4] (19, 88) |
| **STAI-T** (median)  [IQR] | 40.0  [35.0, 46.0] | 40.0  [35.0, 46.0] | 39.0  [34.0, 44.0] | 37.0  [31.5, 42.0] | 39.0  [36.0, 46.0] |
| **Educational status** n (%) |  |  |  |  |  |
| Compulsory education | 25 (10.0) | 12 (9.6) | 7 (12.3) | 1 (2.9) | 5 (15.2) |
| Vocational training | 112 (44.8) | 57 (45.6) | 23 (40.4) | 17 (48.6) | 15 (45.5) |
| University | 105 (42.0) | 51 (40.8) | 26 (45.6) | 15 (42.9) | 13 (39.4) |
| Not specified | 8 (3.2) | 5 (4.0) | 1 (1.8) | 2 (5.7) | 0 (0.0) |
| **Civil status** n (%) |  |  |  |  |  |
| Single | 100 (40.0) | 46 (36.8) | 22 (38.6) | 17 (48.6) | 15 (45.5) |
| Married / in partnership | 149 (59.6) | 79 (63.2) | 34 (59.6) | 18 (51.4) | 18 (54.5) |
| Not specified | 1 (0.4) | 0 (0.0) | 1 (1.8) | 0 (0.0) | 0 (0.0) |
| **Number of children** (median)  (min, max) [IQR] | 1.0  (0, 5) [0.0, 2.0] | 1.0  (0, 4) [0.0, 2.0] | 1  (0, 5) [0.0, 2.0] | 0.5  (0, 5) [0.0, 2.0] | 2.0  (0, 4) [0.0, 3.0] |
| **Personal history of breast biopsy** n (%) |  |  |  |  |  |
| No | 196 (78.4) | 89 (71.2) | 45 (78.9) | 30 (85.7) | 32 (97.0) |
| Yes | 53 (21.2) | 36 (28.8) | 11 (19.3) | 5 (14.3) | 1 (3.0) |
| Unknown | 1 (0.4) | 0 (0.0) | 1 (1.8) | 0 (0.0) | 0 (0.0) |
| **Personal history of breast operation with benign histology** n (%) |  |  |  |  |  |
| No | 226 (90.4) | 114 (91.2) | 49 (86.0) | 31 (88.6) | 32 (97.0) |
| Yes | 23 (9.2) | 11 (8.8) | 7 (12.3) | 4 (11.4) | 1 (3.0) |
| Unknown | 1 (0.4) | 0 (0.0) | 1 (0.8) | 0 (0.0) | 0 (0.0) |
| **Personal history of breast cancer** n (%) |  |  |  |  |  |
| No | 236 (94.4) | 117 (93.6) | 53 (93.0) | 33 (94.3) | 33 (100.0) |
| Yes | 13 (5.2) | 8 (6.4) | 3 (5.3) | 2 (5.7) | 0 (0.0) |
| Unknown | 1 (0.4) | 0 (0.0) | 1 (1.8) | 0 (0.0) | 0 (0.0) |
| **Family history of breast cancer** n (%) |  |  |  |  |  |
| No | 167 (66.8) | 74 (59.2) | 43 (75.4) | 23 (65.7) | 27 (81.8) |
| Yes | 76 (30.4) | 47 (37.6) | 13 (22.8) | 12 (34.3) | 4 (12.1) |
| Unknown | 7 (2.8) | 4 (3.2) | 1 (1.8) | 0 (0.0) | 2 (6.1) |
| **Sonographic breast density*** n (%) |  |  |  |  |  |
| 1 | 23 (9.2) | 10 (8.0) | 5 (8.8) | 3 (8.6) | 5 (15.2) |
| 2 | 79 (31.6) | 35 (28.0) | 23 (40.4) | 16 (45.7) | 5 (15.2) |
| 3 | 113 (45.2) | 62 (49.6) | 22 (38.6) | 10 (28.6) | 19 (57.6) |
| 4 | 34 (13.6) | 17 (13.6) | 7 (12.3) | 6 (17.1) | 4 (12.1) |
| Not specified | 1 (0.4) | 1 (0.8) | 0 (0.0) | 0 (0.0) | 0 (0.0) |
| **Waiting time for biopsy results in days** (median)  [IQR] | 7.0  [3.1, 8.0] | 6.8  [2.9, 7.9] | 7.1  [6.1, 9.9] | 6.7  [3.0, 7.0] | 7.2  [6.8, 10.0] |
| **Histologic diagnosis** n (%) |  |  |  |  |  |
| Benign | 149 (59.6) | 79 (63.2) | 33 (57.9) | 19 (54.3) | 18 (54.5) |
| Malignant | 101 (40.4) | 46 (36.8) | 24 (42.1) | 16 (45.7) | 15 (45.5) |

*according to Madjar et al 2006
