# Supplementary material for: Impact of a brochure and empathetic physician communication on patients’ perception of breast biopsies
Source: Arch Gynecol Obstet. 2023 May 20;308(5):1611–20. doi: 10.1007/s00404-023-07058-w (PMC10520099; doi:10.1007/s00404-023-07058-w)
Supplement: Supplementary file 6 — (DOCX 16 kb) [file 404_2023_7058_MOESM6_ESM.docx]

**Table S2:** Physician’s perception of patient worries, pain and satisfaction

|  | **Study population** | **Control group** | **Intervention group** | **Benign histology** | **Malignant histology** |
| --- | --- | --- | --- | --- | --- |
|  | n=250 | n=125 | n=125 | n=149 | n=101 |
| **Physician’s perception of patient worries, VAS*** |  |  |  |  |  |
| patient anxiety (median)  [IQR] | 3.0  [1.0, 5.0] | 4.0  [2.0, 7.0] | 3.0  [1.0, 4.0] | 3.0  [1.0, 6.0] | 3.0  [1.0, 5.0] |
| patient tension (median)  [IQR] | 4.0  [1.0, 6.0] | 4.0  [2.0, 7.0] | 3.0  [1.0, 5.0] | 3.0  [1.0, 6.0] | 4.0  [1.0, 6.0] |
|  |  |  |  |  |  |
| **Physician’s perception of patient pain, VAS*** |  |  |  |  |  |
| Patient pain (median)  [IQR] | 2.0  [1.0, 4.0] | 2.0  [1.0, 4.0] | 2.0  [1.0, 3.0] | 1.0  [1.0, 3.0] | 2.0  [1.0, 4.0] |
|  |  |  |  |  |  |
| **Physician’s perception of patient satisfaction, VAS**** |  |  |  |  |  |
| Patient satisfaction (median)  [IQR] | 3.0  [1.0, 3.0] | 3.0  [2.0, 4.0] | 1.0  [0.0, 3.0] | 2.0  [1.0, 3.0] | 3.0  [1.0, 3.0] |

* Visual analogue scale (VAS): 0: no worries, pain; 10: maximal worries, pain

**Visual analogue scale (VAS): 0: excellent satisfaction; 10: poor satisfaction
