# Supplementary material for: Impact of a brochure and empathetic physician communication on patients’ perception of breast biopsies
Source: Arch Gynecol Obstet. 2023 May 20;308(5):1611–20. doi: 10.1007/s00404-023-07058-w (PMC10520099; doi:10.1007/s00404-023-07058-w)
Supplement: Supplementary file 7 — (DOCX 16 kb) [file 404_2023_7058_MOESM7_ESM.docx]

**Table S3**: Brochure

|  | **Study**  **population** | **Control**  **group** | **Intervention**  **group** | **Benign**  **histology** | **Malignant**  **histology** |
| --- | --- | --- | --- | --- | --- |
|  | n=250 | n=125 | n=125 | n=149 | n=101 |
| **Time point 2 Control Group** |  |  |  |  |  |
| Would a brochure be helpful? |  |  |  |  |  |
| Would be helpful n (%) | 68 (27.2) | 68 (54.4) |  | 44 (29.5) | 24 (23.8) |
| Not necessary n (%) | 35 (14.0) | 35 (28.0) |  | 22 (14.8) | 13 (12.9) |
| I don’t know n (%) | 21 (8.4) | 21 (16.8) |  | 13 (8.7) | 8 (7.9) |
| I don’t want to respond n (%) | 1 (0.4) | 1 (0.8) |  | 0 (0.0) | 1 (1.0) |
| How helpful would be a brochure? VAS* (median)  [IQR] | 2.0  [1.0, 3.0] | 2.0  [1.0, 3.0] |  | 1.0  [1.0, 3.0] | 3.0  [2.0, 4.0] |
|  |  |  |  |  |  |
| **Time point 2 Intervention Group** |  |  |  |  |  |
| Was the brochure helpful? |  |  |  |  |  |
| Yes, continue to hand it out n (%) | 119 (47.6) |  | 119 (95.2) | 66 (44.3) | 53 (52.5) |
| No, not necessary n (%) | 5 (2.0) |  | 5 (4.0) | 3 (2.0) | 2 (2.0) |
| No response n (%) | 1 (0.4) |  | 1 (0.8) | 1 (0.7) | 0 (0.0) |
| How helpful was the brochure? VAS* (median)  [IQR] | 2.0  [1.0, 3.0] |  | 2.0  [1.0, 3.0] | 1.0  [1.0, 2.0] | 2.0  [1.0, 4.0] |

* Visual analogue scale (VAS): 0: extremely helpful, 10: not helpful at all
